# Supplementary material for: Functional and structural characterization of a flavoprotein monooxygenase essential for biogenesis of tryptophylquinone cofactor
Source: Nat Commun. 2021 Feb 10;12:933. doi: 10.1038/s41467-021-21200-9 (PMC7876137; doi:10.1038/s41467-021-21200-9)
Supplement: Supplementary file 1 — Supplementary Information [file 41467_2021_21200_MOESM1_ESM.pdf]

Supplementary Information

Functional and structural characterization of a flavoprotein monooxygenase essential for biogenesis of tryptophylquinone cofactor

Toshinori Oozeki, Tadashi Nakai, Kazuki Kozakai, Kazuki Okamoto, Shun'ichi Kuroda, Kazuo Kobayashi, Katsuyuki Tanizawa and Toshihide Okajima*

*To whom correspondence should be addressed: T. Okajima (tokajima@sanken.osaka-u.ac.jp)

This file includes:

Supplementary Tables 1 to 3
Supplementary Figs. 1 to 13

Supplementary Table 1. Dissociation constants and association and dissociation rate constants for interactions of wild-type and mutant QhpG with QhpC

QhpG/QhpC	K_d (nM)	Association rate constant (k_a) ($M^{-1}\ s^{-1}$)	Dissociation rate constant (k_d) (s^{-1})
Wild-type/linear	2800 ± 22	$(5.9 \pm 1.7) \times 10^4$	$(1.6 \pm 0.46) \times 10^{-1}$
Wild-type/crosslinked	28 ± 2.3	$(4.9 \pm 0.44) \times 10^5$	$(1.4 \pm 0.0090) \times 10^{-2}$
E42A/crosslinked	65 ± 8.1	$(1.7 \pm 0.16) \times 10^5$	$(1.1 \pm 0.29) \times 10^{-2}$
R47A/crosslinked	1500 ± 490	$(1.5 \pm 0.13) \times 10^4$	$(2.3 \pm 0.52) \times 10^{-2}$
R70A/crosslinked	48 ± 3.6	$(4.7 \pm 0.45) \times 10^5$	$(2.2 \pm 0.04) \times 10^{-2}$
W183F/crosslinked	37 ± 19	$(3.6 \pm 0.68) \times 10^5$	$(1.3 \pm 0.41) \times 10^{-2}$
R314A/crosslinked	78 ± 28	$(1.4 \pm 0.18) \times 10^5$	$(1.1 \pm 0.24) \times 10^{-2}$

Supplementary Table 2. Data collection and crystallographic refinement statistics

Crystal (PDB entry ID)	Hg derivative	Native (7CTQ)
**Data collection**		
Wavelength (Å)	1.0070	0.9
Space group	$P2_1$	$P2_1$
Cell dimensions		
a, b, c (Å),	90.07, 52.20, 101.76,	88.61, 51.56, 101.79,
α, β, γ (°)	90, 99.50, 90	90, 99.77, 90
Resolution (Å)	100 – 2.79 (2.84 – 2.79)[a]	50 – 1.98 (2.10 – 1.98)
$I/\sigma(I)$	13.7 (2.1)	12.7 (2.2)
Redundancy	2.0 (1.9)	3.6 (3.6)
Overall completeness (%)	99.3 (99.8)	98.7 (97.2)
Overall R_{merge} (%)	8.7 (49.4)	6.8 (62.5)
**SIRAS phasing**		
Hg atom sites	2	
Resolution range of data used (Å)	40 – 2.79 (2.81 – 2.79)	
No. reflections	45360	
Figure of merit	0.201 (0.034)	
Phasing power (isomorphous)	0.374 (0.209)	
Phasing power (anomalous)	0.419 (0.112)	
**Refinement**		
Resolution (Å)		50 – 1.98 (2.05 – 1.98)
No. reflections		63142
R_{work} / R_{free} (%)		18.2 (26.8) / 22.2 (34.8)
No. atoms		
Protein		6800
Ligand		192
Water		421
Average temperature factors		
Protein		44.9
Ligand		50.1
Water		44.7
R.m.s. deviation from ideal values		
Bond lengths (Å)		0.0072
Bond angles (°)		0.91
Ramachandran plot statistics (%)		
Residues in favored regions		97.3
Residues in allowed regions		2.5
Outliers		0.2[b]

[a]Values in parentheses refer to the data for the highest resolution shells.

[b]The outlier residues are only Val119 in chains A and B (2/858). Val119 is contained in a short turn (Asp118–Gly120), in which the main chain carbonyl group of Asp118 is hydrogen-bonded to the main chain amide group of Gly120, stabilizing the turn structure with relatively high thermal factors.

Supplementary Table 3. **Oligonucleotides used in this study**

Primer for plasmid construction[a]	Nucleotide sequence[b]	Introduced restriction site or mutation
QhpC (+)	tatacatatgaaacacttgaagcccct	NdeI
QhpC (–)	atatgctagccttgtctttcgggaacacgg	NheI
QhpD (+)	catatgggcgcgctactgaacctggtcgaacgc	NdeI
QhpD (–)	ggatcctcagtgcgccttccgcggggtgatgtagcg	BamHI
QhpG (+)	atatcatatggctgaaccgcgcattgtggtgttgggg	NdeI
QhpG (–)	atatggatcctcaa**a**ccaagccctgctccaacaaccaccg	BamHI
His_6-TEV (+)	catgggcagcagccatcatcatcatcatcacgattacgatatcccaa cgaccgaaaacctttacttccagggcca	NcoI/NdeI
His_6-TEV (–)	tatggccctggaagtaaaggttttcggtcgttgggatatcgtaatcgt gatgatgatgatgatggctgctgcc	NcoI/NdeI
QhpG-E42A (+)	cggtttgcggcggtc**gca**ggggtttcgcagcgg	E42A QhpG
QhpG-E42A (–)	ccgctgcgaaacccc**cgt**gaccgccgcaaaccg	E42A QhpG
QhpG-R47A (+)	cggtcgaaggggtttcgcag**gcg**gtgctggaggggctgcgccac	R47A QhpG
QhpG-R47A (–)	tggcgcagcccctccagcac**cgc**ctgcgaaaccccttcgaccg	R47A QhpG
QhpG-R70A (+)	gcgtgacgaccttcagcgtg**gcg**gtgtgagcgtggtcgagggg	R70A QhpG
QhpG-R70A (–)	cccctcgaccacgctcacac**cgc**cacgctgaaggtcgtcacgc	R70A QhpG
QhpG-W183F (+)	gcctggcggatggctgggcg**ttc**atggcgcgcctggaagacgg	W183A QhpG
QhpG-W183F (–)	ccgtcttccaggcgcgccat**gaa**cgcccagccatccgccaggc	W183A QhpG
QhpG-R314A (+)	ggatcgaacagctgtttttg**gcc**tttgcccggatcgggcggg	R314A QhpG
QhpG-R314A (–)	cccgcccgatccgggcaaa**ggc**caaaaacagctgttcgatcc	R314A QhpG
QhpC-W43F (+)	tccgacccttgctgg**ttc**ccggcccaggtgccg	W43F QhpC
QhpC-W43F (–)	cggcacctgggccgg**gaa**ccagcaagggtcgga	W43F QhpC
pdQhpA[c] (+)	atatcatatgaagccattcacccgaaccgcgc	NdeI
pdQhpA[c] (–)	acatggatcctcaccggatcggcgcatcgacgaa	BamHI
His_6-pdQhpA[c] (+)	ggctcgagccatcaccatcaccatcactcgagcggcgtgacgggc gaggaggtg	His_6-pdQhpA[c]
His_6-pdQhpA[c] (–)	gccgctcgagtgatggtgatggtgatggctcgagcccgcaaggac cggcgcggc	His_6-pdQhpA[c]

[a]Plus and minus signs in parentheses denote sense and antisense strands, respectively.
[b]Restriction sites are underlined. Mismatching nucleotides at the mutated sites are shown in bold.
[c]QhpA of *Pa. denitrificans*.

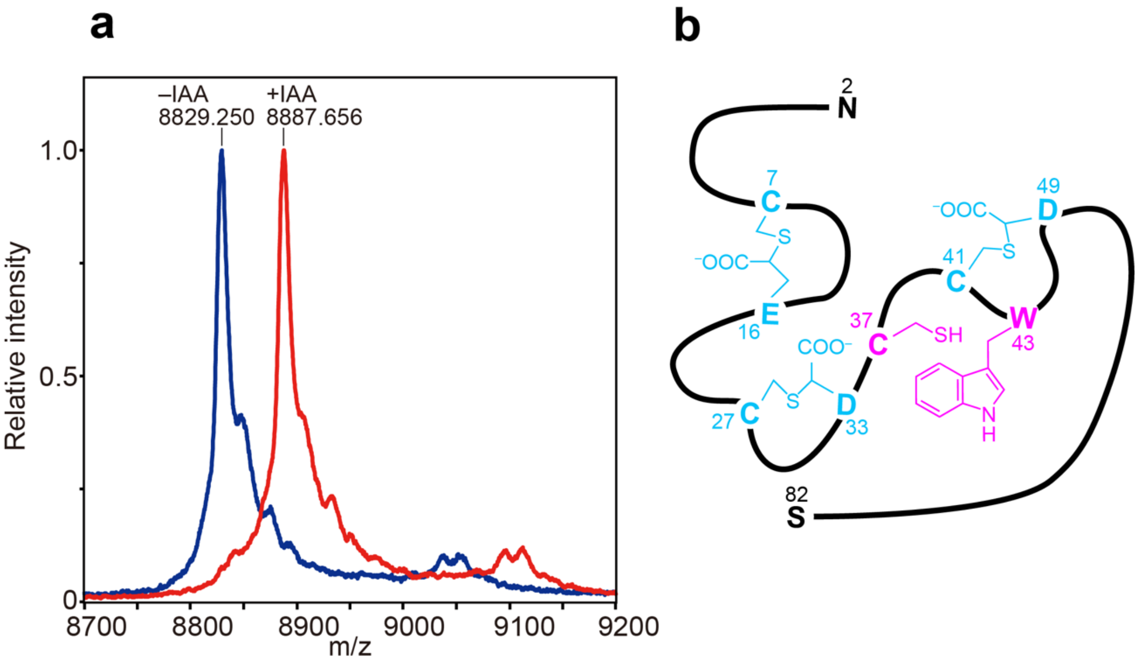

Supplementary Fig. 1. Structural analysis of quinone-less γ-subunit. a, MALDI-TOF mass spectra of the quinone-less γ-subunit (fully processed QhpC) isolated from the *ΔqhpG* strain. −IAA and +IAA indicate before (blue) and after (red) treatment with IAA, respectively. Numbers indicate *m/z* for the peaks. **b**, Schematic drawing of the quinone-less γ-subunit with triple intra-peptidyl sulfur-to-methylene carbon thioether bonds. The CTQ-precursor residues, Trp43 and Cys37, and thioether crosslinks are shown in magenta and cyan, respectively.

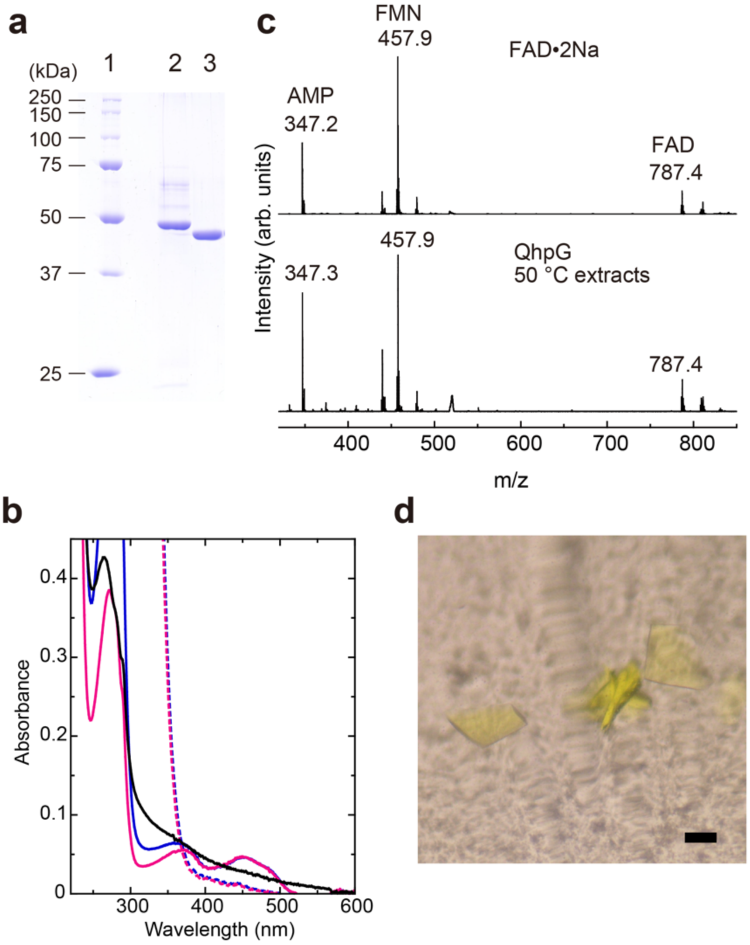

Supplementary Fig. 2. Purification and characterization of QhpG. a, Purification steps monitored by sodium dodecyl sulfate PAGE. About 2-μg protein was applied in each lane. Protein bands were stained with Coomassie Brilliant Blue R-250. Lane 1, precision plus protein dual color standard (Bio-Rad); lane 2, after the first HisTrap HP purification; lane 3, after the TEV proteinase digestion and the second HisTrap HP purification. Sodium dodecyl sulfate PAGE performed in every purification ($n > 10$) showed a similar level of the protein purity. **b**, UV-visible absorption spectra. Magenta curve, purified QhpG (5 μM); blue curve, purified QhpG (5 μM) plus crosslinked QhpC (5 μM); magenta and blue dotted curves, after anaerobic addition of 25 μM dithionite to each sample; black curve, reduced QhpG after removing excess dithionite with a desalting spin column in an anaerobic chamber and adjusted to the same protein concentration with the oxidized QhpG. **c**, MALDI-TOF mass spectrometric analysis of FAD standard (upper panel), and QhpG extract (lower panel). A 1-μl aliquot of the heated extract of QhpG (50 ºC, 10 min) was used for mass spectrometric analysis. Mass

number (*m/z*) and assignments of fragmented FAD are indicated. Intensity is expressed in arbitrary units (arb. units). **d**, A photo of QhpG crystals (scale bar, 100 μm) (n > 10).

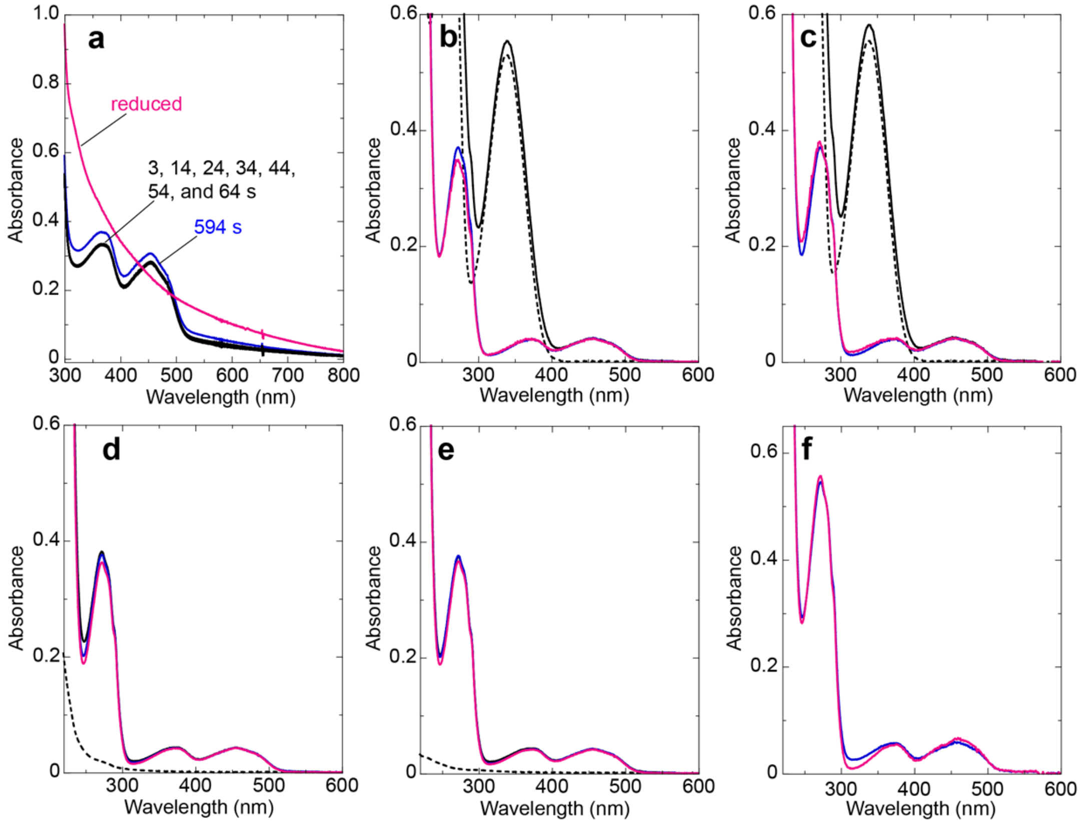

Supplementary Fig. 3. Spectral changes of bound FAD. a, Re-oxidation of reduced FAD by O_2. QhpG (50 μM) was incubated with 250 μM dithionite in 25 mM Tris-HCl, pH 8.0, containing 150 mM NaCl and 10% (w/v) glycerol (buffer C) for 1 h under anaerobic conditions. The reduced QhpG was re-oxidized at 25 ºC by manually mixing with twice volumes of the O_2-satureated buffer C. UV-visible absorption spectra were monitored at indicated times after mixing (black and blue curves). The magenta curve represents the absorption spectrum of reduced QhpG mixed with twice volumes of O_2-depleted buffer C. **b**, **c**, **d**, and **e**, Spectral changes by addition of various reducing reagents. The oxidized form of QhpG (5 μM, blue curve) was incubated with 100 μM NADPH (**b**), NADH (**c**), reduced glutathione (**d**), or dihydrolipoate (**e**) at room temperature for 30 min under anaerobic conditions in buffer C (black curve in each panel). Black dotted and magenta curves represent spectra of the reducing reagent alone and those of QhpG, from which the spectra of reducing reagents were subtracted, respectively. **f**, Spectral changes by addition of free $FADH_2$. A mixture of FAD and $FADH_2$ (about 1:9) was anaerobically prepared by spectral titration with sodium dithionite. QhpG (10 μM) was incubated with the FAD/$FADH_2$ mixture (100 μM) at room temperature for 30 min under anaerobic conditions in buffer C. After removing

the free $FADH_2$ with desalting spin columns, the QhpG absorption spectrum was measured under anaerobic conditions (magenta). The absorption spectrum of the oxidative form of QhpG was also measured after an identical desalting treatment (blue).

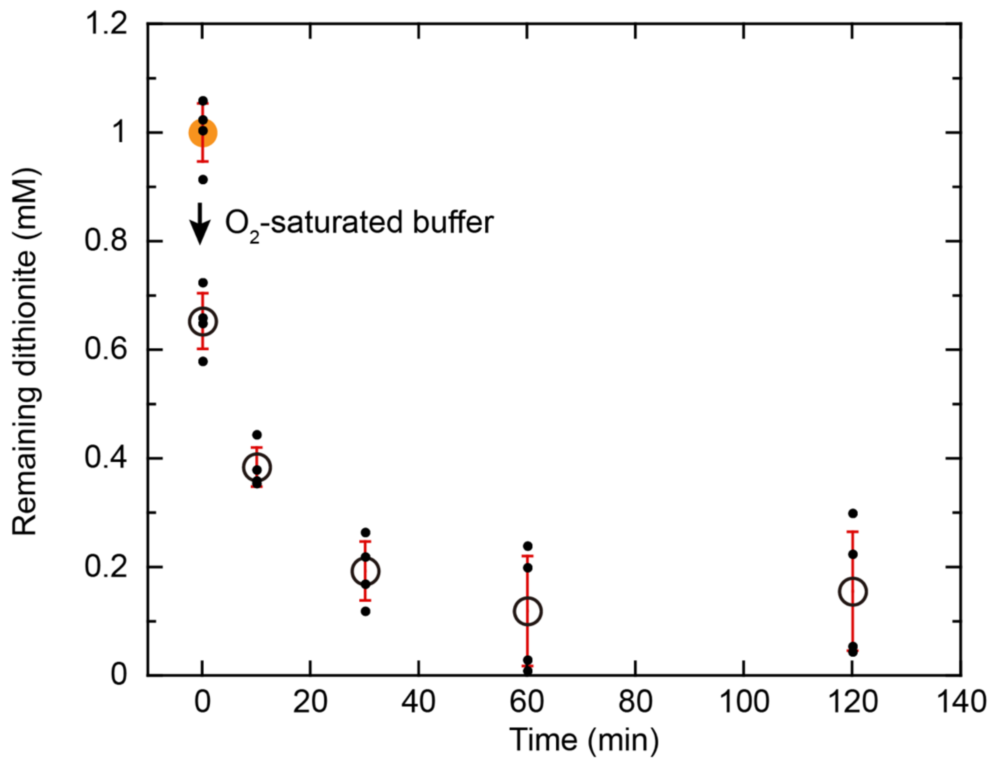

Supplementary Fig. 4. Consumption of dithionite by addition of O_2-saturated buffer. The QhpG-reaction mixture containing 3 mM sodium dithionite (without proteins) was diluted with twice volumes of O_2-saturated buffer C and the remaining dithionite (open circles) was assayed by measuring spectral changes of an anaerobic solution of free FAD using an aliquot withdrawn at appropriate time intervals. Initial concentrations of dithionite (orange closed circle) were determined after addition of twice volumes of O_2-depleted buffer C. Data are presented as mean values (closed and open circles) ± s.d. (error bars) of n = 4 measurements (black dots) from two independent experiments.

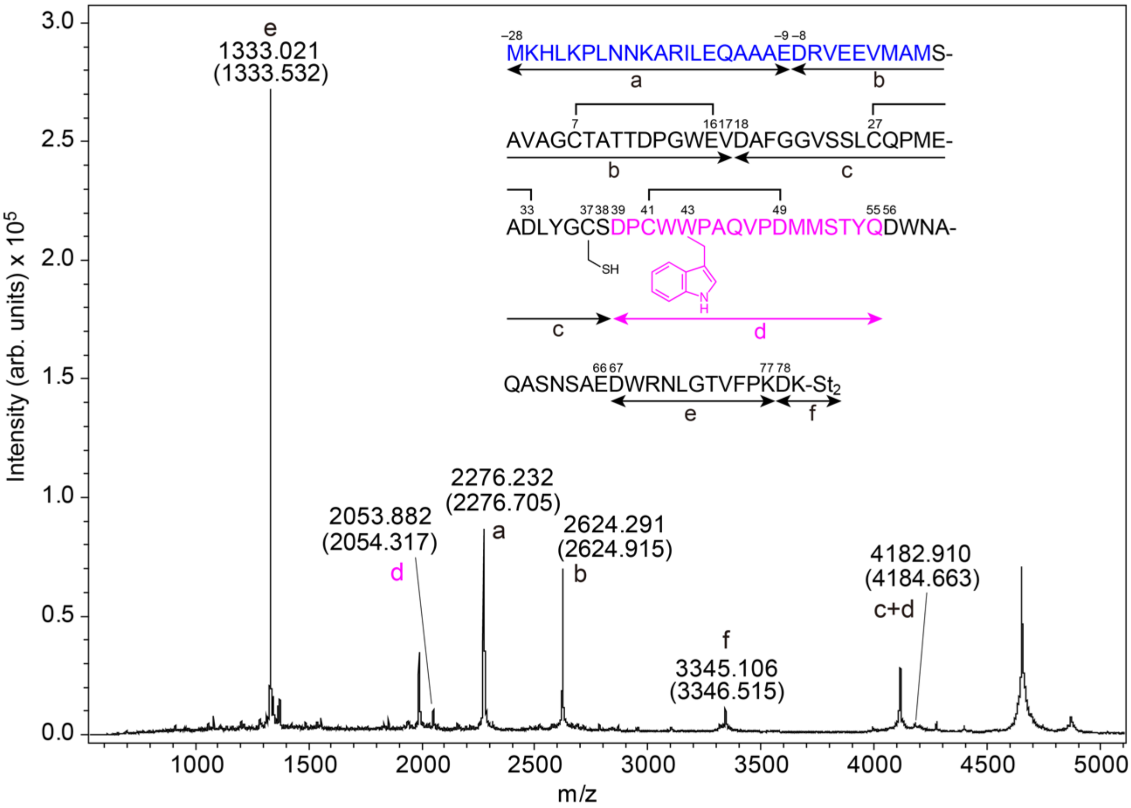

Supplementary Fig. 5. MALDI-TOF mass spectrometric analysis of crosslinked QhpC digested with Asp-N. The observed and calculated (in parentheses) mass values (*m/z*) are indicated with an assigned fragment code. Fragment d (shown in magenta) contains the CTQ-precursor Trp43. Intensity is expressed in arbitrary units (arb. units). *Inset*: schematic representation of peptide fragments produced by Asp-N digestion. The leader peptide and fragment d are shown in blue and magenta, respectively.

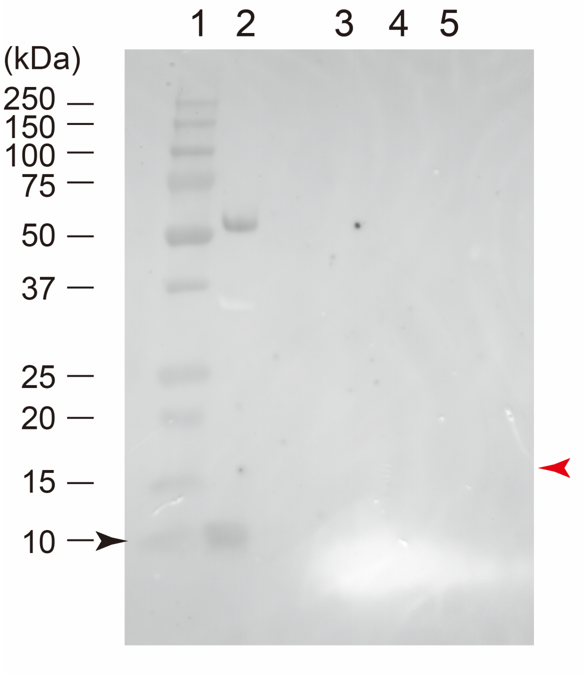

Supplementary Fig. 6. Redox-cycling quinone staining of QhpG reaction product. The precipitated QhpG reaction product was subjected to sodium dodecyl sulfate PAGE and blotted onto a membrane for redox-cycling quinone staining. Lane 1, precision plus protein dual color standard (Bio-Rad); lane 2, purified QHNDH (0.5 μg); lanes 3–5, QhpG reaction products (total protein, ~20 μg) before (lane 3) and after the reaction in the presence of 1 mM sodium dithionite (lane 4) or 1 mM NADPH (lane 5). Black and red arrowheads indicate approximate positions of the γ-subunit (~9 kDa) (positive control) and crosslinked QhpC with the leader peptide (~15 kDa), respectively. A positive band of ~60 kDa in lane 2 is probably derived from hemes contained in the α-subunit of QHNDH. The experiments repeated twice independently gave similar results.

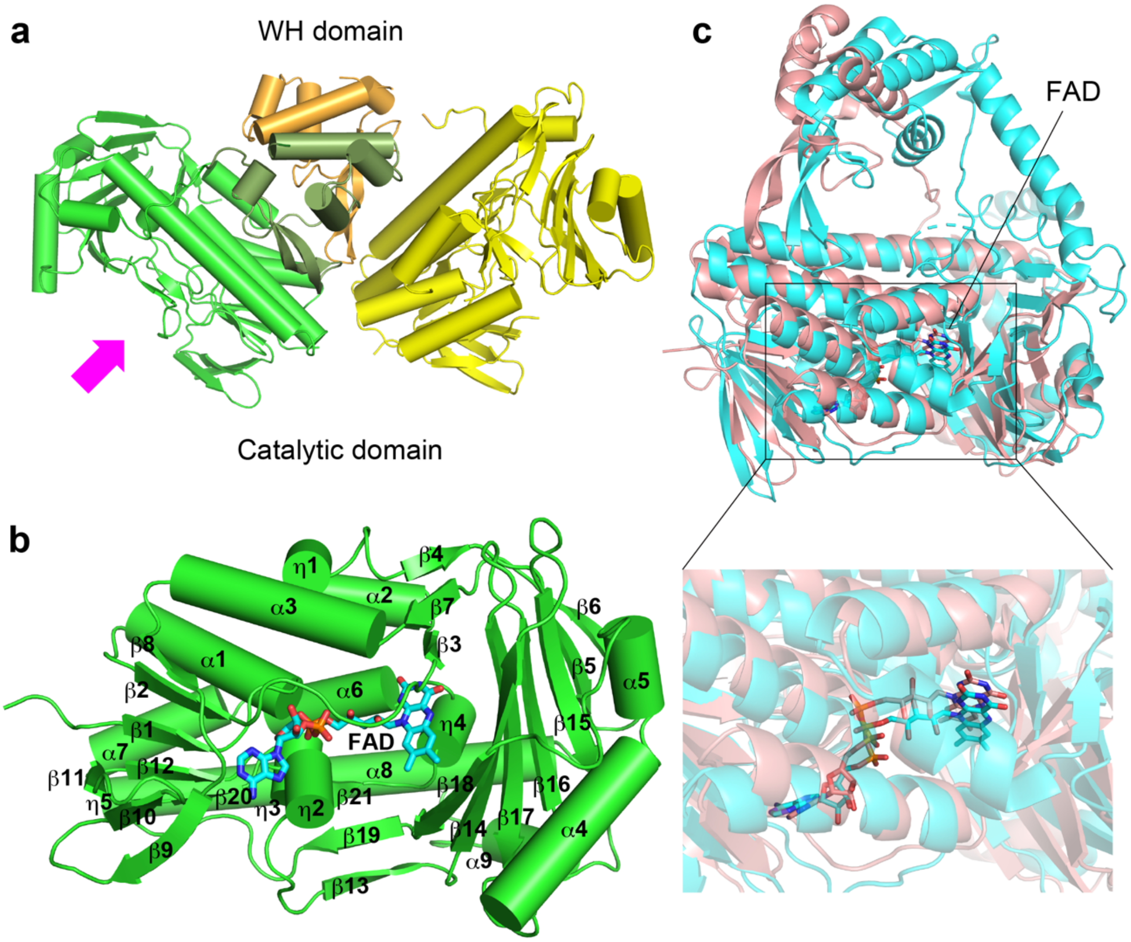

Supplementary Fig. 7. Schematic drawings of the asymmetric unit in QhpG crystal and the FAD-binding region. a, An asymmetric unit showing inter-domain interactions between the two monomers, colored green and yellow for the catalytic domains and moss green and orange for respective WH domains. **b**, The FAD-binding region of the catalytic domain viewed form the direction of the magenta arrow shown in **a**. The secondary structural elements assigned to the main chain structure (*cf.* Supplementary Fig. 8) are labeled. FAD is bound between two lobes; one consisting of the central 5-stranded parallel β-sheet (β1, β2, β8, β12, β20), three 3-stranded anti-parallel β-sheets (β3, β4, β7; β9–β11; β13, β19, β21), and 10 helices (α1–α3, α6–α8, η1–η3, η5; where η means a 3_{10}-helix) for the ADP moiety and the other consisting of a mixed 7-stranded β-sheet (β5, β6, β14–β18) in the core layer and 4 helices (α4, α5, α9, η4) in the surface layer for the isoalloxazine ring. **c**, Structural comparison of QhpG (brown) with SvCmlS (cyan) with an enlarged view of the FAD-binding region. Proteins and the bound FAD were drawn by cartoon and stick models, respectively.

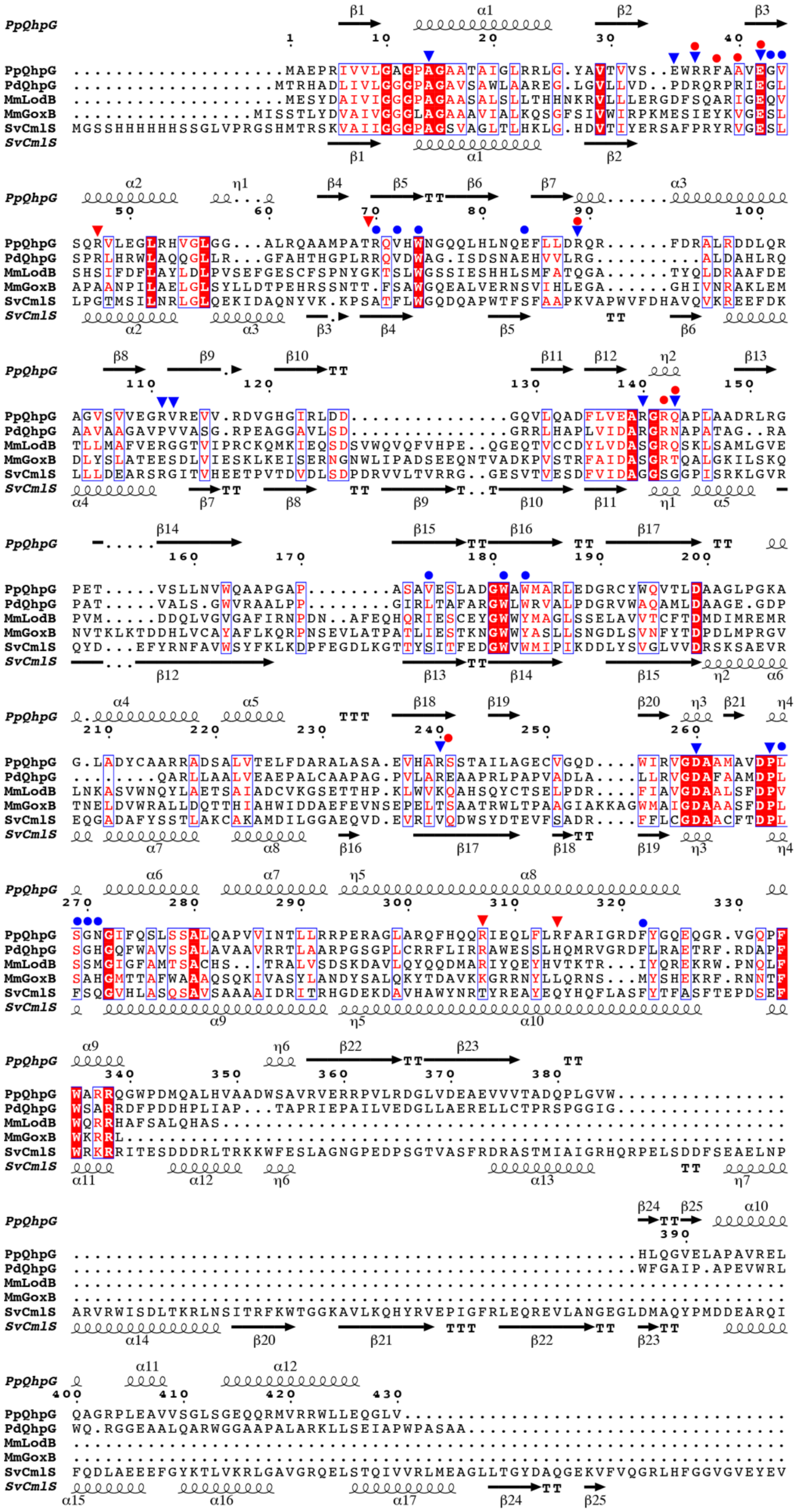
PpQhpG ...................MAEPRIVVLGAGPAGAATAIGLRRLG.YAVTVVS..EWRRFAAVEGV
PdQhpG ..................MTRHADLIVLGGGPAGAVSAWLAAREG.LGVLLVD..PDRQRPRIEGL
MmLodB ..................MESYDAIVIGGGPAGAASALSLLTHHNKRVLLLERGDFSQARIGEQV
MmGoxB ................MISSTLYDVAIVGGGLAGAAAVIALKQSGFSIVWIRPKMESIEYKVGESL
SvCml5 MGSSHHHHHHSSGLVPRGSHMTRSKVAIIGGGPAGSVAGLTLHKLG.HDVTIYERSAFPRYRVGESL
PpQhpG SQRVLEGLRHVGLGG...ALRQAAMPATRQVHWNGQQLHLNQEFLLDRQR......FDRALRDDLQR
PdQhpG SPRLHRWLAQQGLLR...GFAHTHGPLRRQVDWAG.ISDSNAEHVVLRG..........ALDAHLRQ
MmLodB SHSIFDFLAYLDLPVSEFGESCFSPNYGKTSLWGSSIESHHLSMFATQGA......TYQLDRAAFDE
MmGoxB APAANPILAELGLSYLLDTPEHRSSNTT.FSAWGQEALVERNSVIHLEGA......GHIVNRAKLEM
SvCml5 LPGTMSILNRLGLQEKIDAQNYVK.KPSATFLWGQDQAPWTFSFAAPKVAPWVFDHAVQVKREEFDK
PpQhpG AGVSVVEGRVREVV.RDVGHGIRLDD..............GQVLQADFLVEARGRQAPLAADRLRG
PdQhpG AAVAAGAVPVVASG.RPEAGGAVLSD..............GRRLHAPLVIDARGRNAPATAG..RA
MmLodB TLLMAFVERGGTVIPRCKQMKIEQSDSVWQVQFVHPE.QGEQTVCCDYLVDASGRQSKLSAMLGVE
MmGoxB DLYSLATEESDLVIESKLKEISERNGNWLIPADSEEQNTVADKPVSTRFAIDASGRTQALGKILSKQ
SvCml5 LLLDEARSRGITVHEETPVTDVDLSDPDRVVLTVRRG..GESVTVESDFVIDAGGSGGPISRKLGVR
PpQhpG PET.....VSLLNVWQAAPGAP........ASAVESLADGWAWMARLEDGRCYWQVTLDAAGLPGKA
PdQhpG PAT.....VALS.GWVRAALPP........GIRLTAFARGWLWRVALPDGRVWAQAMLDAAGE.GDP
MmLodB PVM.....DDQLVGVGAFIRNPDN..AFEQHQRIESCEYGWWYMAGLSSELAVVTCFTDMDIMREMR
MmGoxB NVTKLKTDDHLVCAYAFLKQRPNSEVLATPATLIESTKNGWWYASLLSNGDLSVNFYTDPDLMPRGV
SvCml5 QYD...EFYRNFAVWSYFKLKDPFEGDLKGTTYSITFEDGWVWMIPIKDDLYSVGLVVDRSKSAEVR
PpQhpG G.LADYCAARRADSALVTELFDARALASA.EVHARSSTAILAGECVGQD....WIRVGDAAMAVDPL
PdQhpG ........QARLLAALVEAEPALCAAPAG.PVLAREAAPRLPAPVADLA....LLRVGDAFAAMDPL
MmLodB LNKASVWNQYLAETSAIADCVKGSETTHP.KLWVKQAHSQYCTSELPDR....FIAVGDAALSFDPV
MmGoxB TNELDVWRALLDQTTHIAHWIDDAEFEVNSEPELTSAATRWLTPAAGIAKKAGWMAIGDAAASFDPL
SvCml5 EQGADAFYSSTLAKCAKAMDILGGAEQVD.EVRIVQDWSYDTEVFSADR....FFLCGDAACFTDPL
PpQhpG SGNGIFQSLSSALQAPVVINTLLRRPERAGLARQFHQQRIEQLFLRFARIGRDFYGQEQGR.VGQPF
PdQhpG SGHGQFWAVSSALAVAAVRRTLAARPGSGPLCRRFLIRRAWESSLHQMRVGRDFLRAETRF.RDAPF
MmLodB SSMGIGFAMTSACHS...TRALVSDSKDAVLQYQQDMARIYQEYHVTKTR...IYQREKRW.PNQLF
MmGoxB SAHGMTTAFWAAAQSQKIVASYLANDYSALQKYTDAVKKGRRNYLLQRNS...MYSHEKRF.RNNTF
SvCml5 FSQGVHLASQSAVSAAAAIDRITRHGDEKDAVHAWYNRTYREAYEQYHQFLASFYTFASFTEPDSEF
PpQhpG WARRQGWPDMQALHVAADWSAVRVERRPVLRDGLVDEAEVVVTADQPLGVW...............
PdQhpG WSARRDFPDDHPLIAP...TAPRIEPAILVEDGLLAERELLCTPRSPGGIG...............
MmLodB WQRRHAFSALQHAS...
MmGoxB WKRRL..
SvCml5 WRKRRITESDDDRLTRKKWFESLAGNGPEDPSGTVASFRDRASTMIAIGRHQRPELSDDFSEAELNP
PpQhpG ....HLQGVELAPAVREL
PdQhpG ....WFGAIP.APEVWRL
MmLodB ....
MmGoxB ....
SvCml5 ARVRWISDLTKRLNSITRFKWTGGKAVLKQHYRVEPIGFRLEQREVLANGEGLDMAQYPMDDEARQI
PpQhpG QAGRPLEAVVSGLSGEQQRMVRRWLLEQGLV....................................
PdQhpG WQ.RGGEAALQARWGGAAPALARKLLSEIAPWPASAA..............................
MmLodB ....
MmGoxB ....
SvCml5 FQDLAEEEFGYKTLVKRLGAVGRQELSTQIVVRLMEAGLLTGYDAQGEKVFVQGRLHFGGVGVEYEV

Supplementary Fig. 8. Multiple sequence alignment and secondary structure assignment of QhpG orthologs and homologs. Amino acid sequences of QhpG orthologs from *Ps. putida* IFO 15366 (PpQhpG, NCBI RefSeq: LC575123 [https://www.ncbi.nlm.nih.gov/nuccore/LC575123.1/]) and *Pa. denitrificans* Pd1222 (PdQhpG, WP_011747997 [https://www.ncbi.nlm.nih.gov/protein/WP_011747997.1]) and homologs; LodB from *Marinomonas mediterranea* MMB-1 (MmLodB, WP_013661795 [https://www.ncbi.nlm.nih.gov/protein/WP_013661795.1]), GoxB from *Marinomonas mediterranea* (MmGoxB, WP_013660822 [https://www.ncbi.nlm.nih.gov/protein/WP_013660822.1]), and CmlS from *Streptomyces venezuelae* (SvCmlS, AAK08979 [https://www.ebi.ac.uk/ena/browser/view/AAK08979]) were aligned using the program Clustal W[1]. Secondary structural elements in the crystal structure of QhpG and SvCmlS were assigned using the program DSSP[2], and are shown above and below each sequence, respectively. Residues, involved in binding of FAD or found in its vicinity, are shown in inverted blue triangles. Residues forming *re*-face and *si*-face channels are shown in blue and red circles, respectively. Positively charged residues locating near the *re*-face channel entrance are shown in inverted red triangles. The figure was drawn using the program ESPript[3].

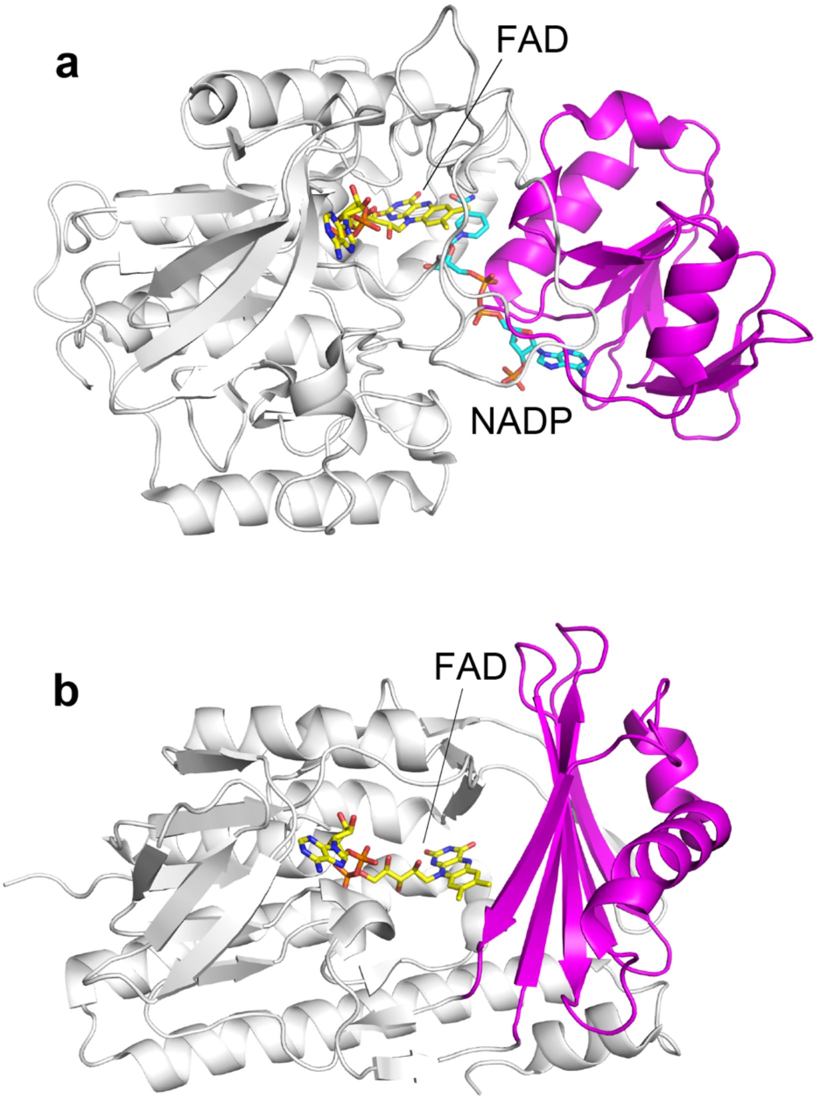

Supplementary Fig. 9. Structural comparison of QhpG and an FAD-dependent monooxygenase requiring NADPH. a, X-ray crystal structure of the flavoprotein monooxygenase from *Schizosaccharomyces pombe* (PDB ID: 2GV8)[4] is shown by a cartoon model with the NADPH-binding domain (Tyr180–Gly289) colored magenta and the bound FAD (yellow) and NADPH (cyan) shown by stick models. **b**, The crystal structure of QhpG (this study) is shown by a cartoon model colored magenta for the region (Glu155–Thr243) besides the FAD-binding domain and the bound FAD shown by a stick model.

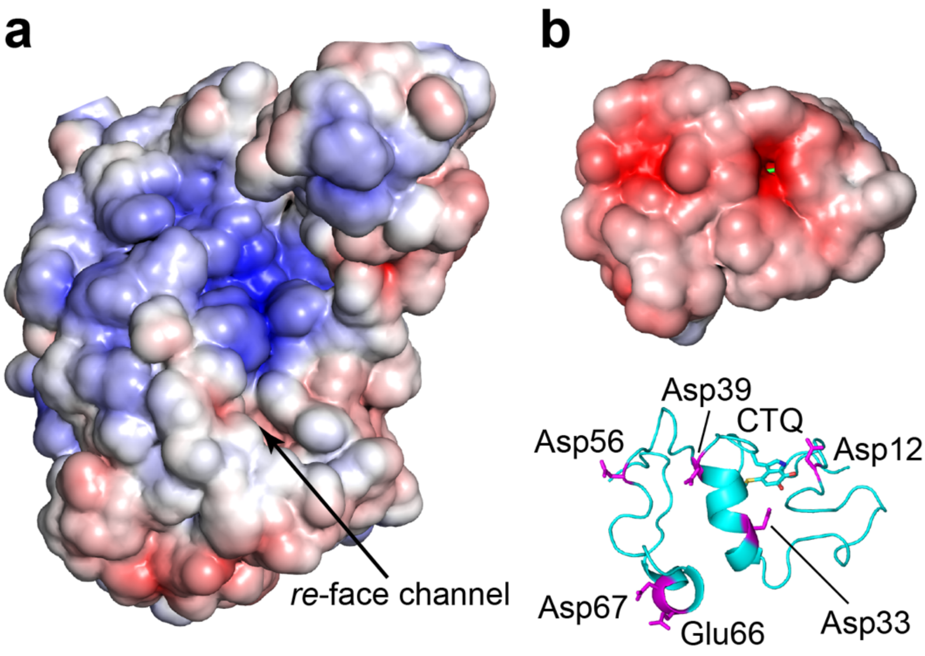

Supplementary Fig. 10. Electrostatic surface potentials of QhpG and γ-subunit of QHNDH. Electrostatic potentials mapped onto the molecular surfaces of QhpG (**a**) and γ-subunit of QHNDH (**b**), colored in gradation from red (−5 *kT*) to blue (+5 *kT*), where *k* is the Boltzmann constant, and *T* is the absolute temperature, based on the calculation by PyMOL (Schrödinger Inc. New York, USA) and APBS[5]. In panel **b**, the γ-subunit structure (cartoon model, cyan) extracted from the *Ps. putida* QHNDH structure (1JMX) is also shown with surface Asp/Glu residues (magenta) and CTQ in stick models.

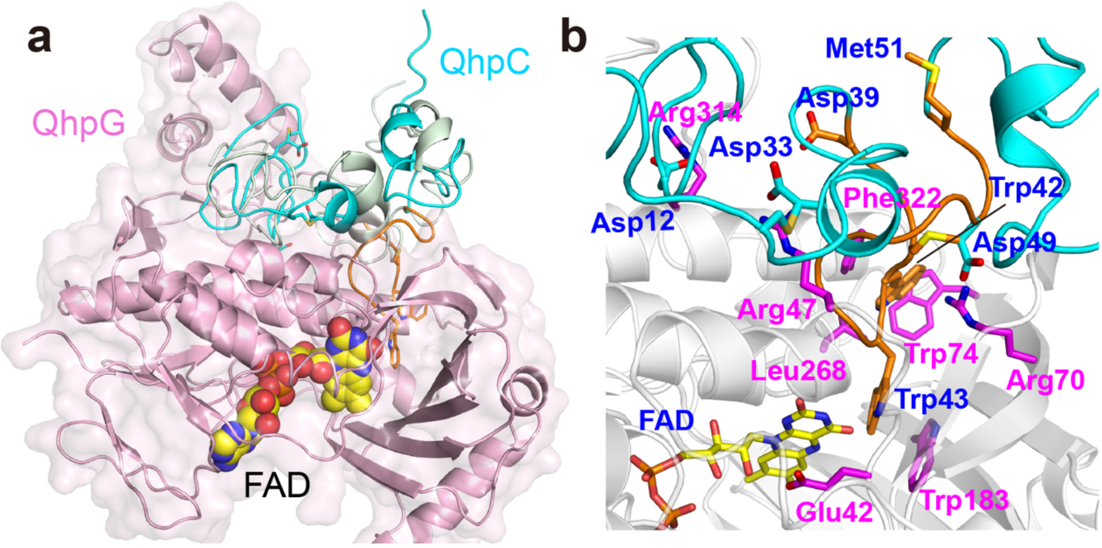

Supplementary Fig. 11. Crosslinked QhpC-QhpG docking model. a, Overall structure of the docking model. A cartoon model of QhpG is colored light magenta with a spherical model of bound FAD and transparent molecular surfaces. The initial docking model of γ-subunit obtained with ZDOCK[6] is shown by pale green. The crosslinked QhpC (without the leader peptide) obtained by Coot[7]-based manual building followed by energy minimization is shown by orange for the Asp39–Met51 loop with the side chains of Trp42 and Trp43 and by cyan for the other region. The thioether bonds between Cys and Asp/Glu residues are shown in stick models. **b**, An enlarged view of the crosslinked QhpC-QhpG docking model. QhpG is depicted by a transparent white cartoon model with the side chains (magenta stick models) that are involved in the interactions with the crosslinked QhpC. FAD is also shown as a stick model. The bound QhpC is presented by the same colors used in panel **a** with the side chains involved in the interaction with QhpG and that of the CTQ precursor Trp43 (orange stick models).

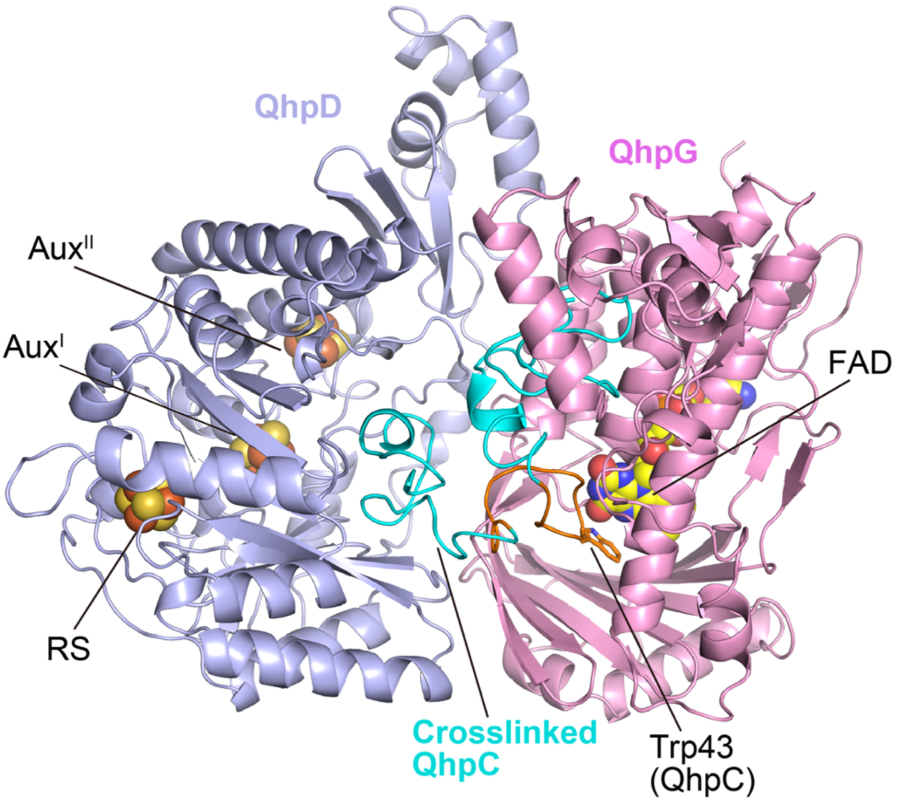

Supplementary Fig. 12. Docking model of the QhpCDG ternary complex. A homology model of QhpD from *Ps. putida* (light blue) was docked onto the model of the QhpCG binary complex (Supplementary Fig. 11) using ZDOCK[6]. Cartoon models of QhpD (light blue), QhpG (light magenta), and crosslinked QhpC (cyan; the Asp39–Met51 loop and the side chain of Trp43, orange) are depicted with FAD in QhpG and three [Fe_4S_4] clusters (RS, Aux^{I}, and Aux^{II}) in QhpD[8] shown by spherical models.

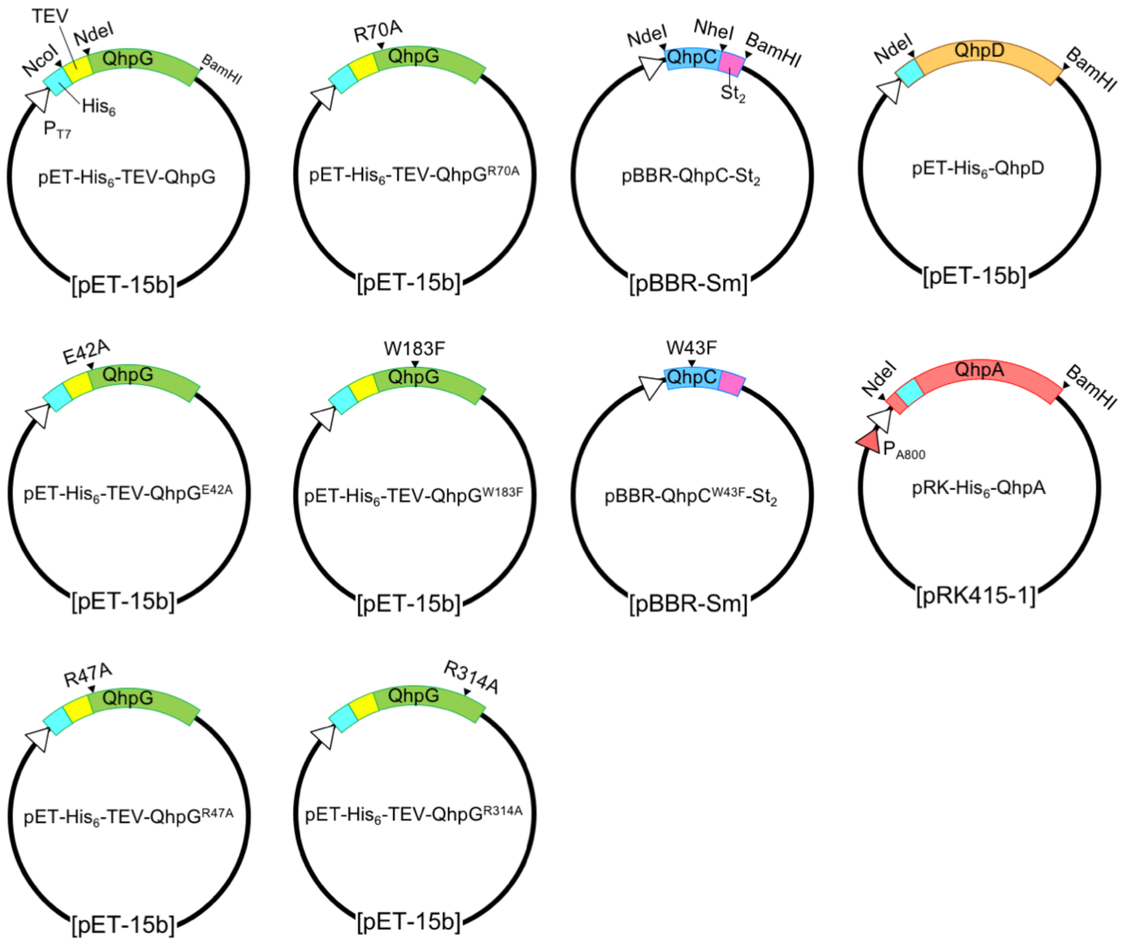

Supplementary Fig. 13. Schematic structures of the plasmids used in this study. In all plasmids, only the restriction sites used for plasmid construction are indicated. His_6-tag, TEV cleavage site (TEV), and St_2-tag are shown by cyan, yellow, and pink bars, respectively. A T7 promoter (P_{T7}) and a promoter region of the *qhp* operon (P_{A800})[9] are depicted by white and red triangles, respectively. Coding regions for QhpA from *Pa. denitrificans*, QhpC (wild-type and W43F), QhpD, and QhpG (wild-type, E42A, R47A, R70A, W183F, and R314A) are colored red, sky blue, orange, and green, respectively, with vectors used for construction (pRK415-1, pET-15b, or pBBR1).

References

1. Thompson, J. D., Higgins, D. G. & Gibson, T. J. CLUSTAL W: Improving the sensitivity of progressive multiple sequence alignment through sequence weighting, position-specific gap penalties and weight matrix choice. *Nucleic Acids Res.* **22**, 4673–4680 (1994).
2. Kabsch, W. & Sander, C. Dictionary of protein secondary structure: pattern recognition of hydrogen-bonded and geometrical features. *Biopolymers* **22**, 2577–2637 (1983).
3. Gouet, P., Courcelle, E., Stuart, D. I. & Metoz, F. ESPript: analysis of multiple sequence alignments in PostScript. *Bioinformatics* **15**, 305–308 (1999).
4. Eswaramoorthy, S., Bonanno, J. B., Burley, S. K. & Swaminathan, S. Mechanism of action of a flavin-containing monooxygenase. *Proc. Natl. Acad. Sci. U. S. A.* **103**, 9832–9837 (2006).
5. Jurrus, E. et al. Improvements to the APBS biomolecular solvation software suite. *Protein Sci.* **27**, 112–128 (2018).
6. Pierce, B. G. et al. ZDOCK server: interactive docking prediction of protein-protein complexes and symmetric multimers. *Bioinformatics* **30**, 1771–1773 (2014).
7. Emsley, P., Lohkamp, B., Scott, W. G. & Cowtan, K. Features and development of Coot. *Acta Crystallogr. Sect. D Biol. Crystallogr.* **D66**, 486–501 (2010).
8. Nakai, T. et al. The radical *S*-adenosyl-L-methionine enzyme QhpD catalyzes sequential formation of intra-protein sulfur-to-methylene carbon thioether bonds. *J. Biol. Chem.* **290**, 11144–11166 (2015).
9. Nakai, T. et al. Identification of genes essential for the biogenesis of quinohemoprotein amine dehydrogenase. *Biochemistry* **53**, 895–907 (2014).
