# Supplementary material for: Functional and structural characterization of a flavoprotein monooxygenase essential for biogenesis of tryptophylquinone cofactor
Source: Nat Commun. 2021 Feb 10;12:933. doi: 10.1038/s41467-021-21200-9 (PMC7876137; doi:10.1038/s41467-021-21200-9)
Supplement: Supplementary file 4 — Description of Additional Supplementary Files [file 41467_2021_21200_MOESM4_ESM.docx]

**Description of Additional Supplementary Files**

File name: Supplementary Movie 1

Description: In the docking model of QhpG and crosslinked QhpC, the molecular surface of QhpG is drawn in magenta with the bound FAD shown by a spherical model. The crosslinked QhpC is first depicted by a cyan cartoon model, in which the crosslinking residues (Cys and Asp or Glu) and the side chains of Trp42 and Trp43 are depicted by orange and cyan stick models, respectively, then its surface is drawn in cyan. The docking model is rotated around the bound FAD of QhpG. In the enlarged view, the residues that are predicted to be involved in the interactions between QhpG and crosslinked QhpC are also shown by stick models.
